# Physiological-Social Scores in Predicting Outcomes of Prehospital Internal Patients

**DOI:** 10.1155/2014/312189

**Published:** 2014-09-14

**Authors:** Abbasali Ebrahimian, Hesam Seyedin, Roohangiz Jamshidi-Orak, Gholamreza Masoumi

**Affiliations:** ^1^School of Nursing and Allied Medical Sciences, Semnan University of Medical Sciences, Semnan, Iran; ^2^Department of Health Services Management, School of Health Management and Information Sciences, Iran University of Medical Sciences, Tehran, Iran; ^3^School of Health Management and Information Sciences, Iran University of Medical Sciences, No. 6, Rashid Yasemi Street, Vali-e-asr Avenue, Tehran 1995614111, Iran; ^4^Emergency Department, 7th Tir Hospital, Iran University of Medical Sciences, Tehran, Iran

## Abstract

The physiological-social modified early warning score system is a newly developed instrument for the identification of patients at risk. The aim of this study was to investigate the feasibility of using the physiological-social modified early warning score system for the identification of patients that needed prehospital emergency care. This prospective cohort study was conducted with 2157 patients. This instrument was used as a measure to detect critical illness in patients hospitalised in internal wards. Judgment by an emergency medicine specialist was used as a measure of standard. Data were analyzed by using receiver operating characteristics curves and the area under the curve with 95% confidence interval. The mean score of the physiological-social modified early warning score system was 2.71 ± 3.55. Moreover, 97.6% patients with the score ≥ 4 needed prehospital emergency services. The area under receiver operating characteristic curve was 0.738 (95% CI = 0.708–0.767). Emergency medical staffs can use PMEWS ≥ 4 to identify those patients hospitalised in the internal ward as at risk patients. The physiological-social modified early warning score system is suggested to be used for decision-making of emergency staff about internal patients' wards in EMS situations.

## 1. Introduction

Emergency medical services (EMS) are at the front line of meeting patients' needs out of hospital environments. Emergency medical staffs are main persons who impact the decision for patients' transference to inpatient healthcare settings. Judgments by emergency medical staffs in prehospital situations are rarely evidence-based and are mainly rooted in subjective data and staffs' level of education and past experiences [1–3]. Sometimes, their decisions may result in inappropriate transfer of patients to hospitals by ambulance or leaving patients on their own, while they must be transferred to the hospital [4].

Unnecessary transfer and improper or excessive use of prehospital emergency services are unresolved issues in the EMS [5]. There are some reports about avoidable use of prehospital care in several countries, including the USA, UK, and Iran [6–8]. Improper use of prehospital EMS leads to wasting limited resources, idling real emergency patients to receive appropriate care, increasing morbidity and mortality rate of patients with conditions that require emergency services, low-quality performance, and job dissatisfaction in prehospital emergency staffs [5]. Therefore, establishing a control mechanism can reduce unnecessary patient transfer and inappropriate use of EMS [9].

There are some well-known methods to determine the severity of illness in various conditions of hospital environments such as the early warning scoring (EWS) systems [10–12]. The EWS systems commonly use vital sign criteria to warn medical staffs of patients' condition [13, 14]. The aim of such systems is fast recognition of critical patients to trigger appropriate responses [15–17]. In the last decade, a few studies have been carried out related to prehospital patient harm events [18]. Moreover, in the prehospital environment, there are no suitable scoring systems for EMS to support decision-making regarding patients' transfer to hospital [19]. Thus, the development of a valid and reliable early warning score system is important to recognise at risk patients in the prehospital environment [20]. The modified early warning score (MEWS) is one of the simple scoring systems that can be used in emergency situations [21, 22].

In the previous study [8], the MEWS was applied to identify patients with medical (nontraumatic) conditions in prehospital emergency environments. It was shown that the MEWS was efficient in the identification of critically ill patients and its sensitivity was in an acceptable level. However, its sensitivity was mentioned to be low and was not efficient enough to determine doubtful patients who need prehospital emergency care [8]. A few studies have investigated the applicability of physiological-social modified scoring system (PMEWS) for patients hospitalised in the internal ward in prehospital care [4, 23]. Therefore, in this study, the authors used the PMEWS to identify the patients with medical (nontraumatic) conditions in prehospital emergency situations. The PMEWS also contain some social norms and is able to examine various variables in comparison to the MEWS. The aim of this study was to investigate the feasibility of using the PMEWS for the identification of patients that needed prehospital emergency care.

## 2. Method

This is a prospective, cohort study. All patients from 23 July to 22 September 2012 following a complaint of an internal pathology problem transferred to a hospital by the EMS in Tehran city were invited to participate in this study. We included only those patients complaining from internal diseases. Trauma patients, patient's less than 12 years of age, and pregnant and mentally ill patients were excluded from this study.

To access the participants, two sites out of four regions of emergency prehospital activities in Tehran were randomly selected. The data collection tool was a form composed of two parts. The first part consisted of the physiological parameters including systolic blood pressure, heart rate, respiratory rate, oxygen saturation percent, temperature, and status of consciousness (APVU; alert, verbal response, and no response to painful stimuli). The second part consisted of the data on social isolation as living alone or having no fixed abode, chronic diseases, age, and performance status. These factors were combined to produce a physiological-social modified early warning score (Table 1) derived from previous studies [19, 23].

Prehospital emergency technicians filled in the patient's transference routine form and at the same time completed the PMEWS standards form. Then they classified the patients into two groups:

1. patients who really required transfer to hospital,
2. patients who did not require the EMS response.

This was done based on the emergency medicine specialist judgment as a gold standard in this study. The ROC curve was used to demonstrate the sensitivity and specificity of PMEWS form.

## 3. Results

A total of 2305 participants entered the study, and 2157 forms were properly completed (response rate 93.6%). The average and standard deviation of the patients' age were 50.58 ± 22.15. Also, 44.7% of the participants were men and 55.3% of them were women. Mean and standard deviation of physiological scores of transferred patients, social scores, and the PMEWS were reported to be 1.97 ± 2.86, 0.75 ± 1.16, and 2.71 ± 3.55, respectively (Table 2).

According to emergency medicine specialists' judgements, 68.4% of the patients who were transported to the hospital required emergency medical services and 31.6% of them did not require any emergency treatments (Table 2).

**Figure 1:**
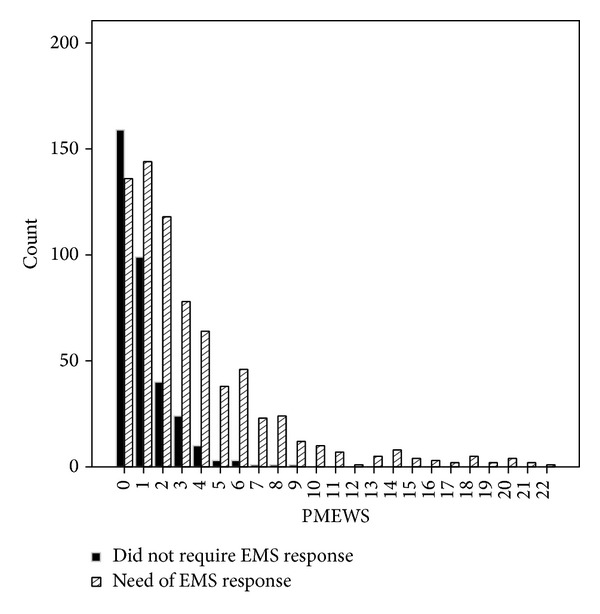
Distribution of the PMEWS in relation to final disposal.

**Figure 2:**
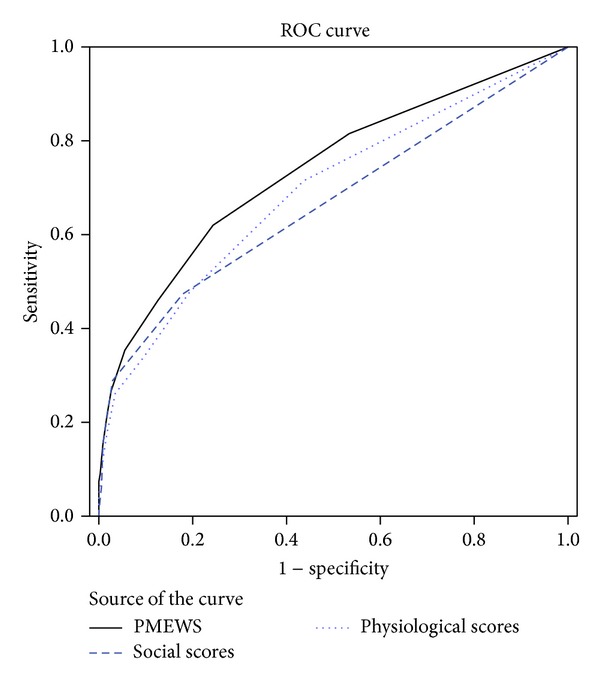
ROC curve for physiological, social, and PMEWS scores as predictors of need of the EMS response.

The results showed that with the increase in PMEWS scores the need for patients to be transferred is increased. 97.6% of patients with the score of 4 or more and all of the patients with the score of 10 needed emergency transport (Figure 1). The area under receiver operating characteristic curve (AUROC) using the PMEWS score as a discriminator for requiring emergency transportation was 0.738 (95% CI = 0.708–0.767) and for physiological score was 0.692 (95% CI = 0.660–0.724) and for social score was 0.667 (95% CI = 0.635–0.699) (Figure 2).

## 4. Discussion

Designing a system to identify patients in need of receiving prehospital emergency care has been emphasized in previous studies [8, 11, 19, 24]. Our findings revealed that when the PMEWS score is equal to or lower than 4, the PMEWS can predict the seriousness of critically-ill patients' need for receiving the EMS.

The sensitivity of this tool was mentioned to be 97.65, using 4 as a cutoff point. This cutoff point in the Fullerton et al. study was three [24] and two in Challen and Walter study [19]. The differences in cutoff points in these studies are due to a variety of research communities. The skills of prehospital emergency medical technicians may vary in different studies. Another reason could be the variation of the quality in hospital services and physicians' judgment in these hospitals. It is suggested that every society should use the PMEWS with an appropriate cutoff point based on its demographic and cultural components. Discovery of this cutoff point requires scientific research with a sufficient sample size.

The area under AUROC curve using the score as a discriminator for admission was 0.767. This finding is close to the area under AUROC curve in the studies by Challen and Walter (0.710), Fullerton et al. (0.799), and Duckitt et al. (0.720) [19, 24, 25]. Limited variables in the PMEWS might be an important cause for PMEWS inability to identify doubtful patients that need emergency care. The ROC curves showed that the use of PMEWS compared to using MEWS and social scores is stronger in predicting the need for patients to be transported by the EMS. Adding some variables to the PMEWS is suggested to increase its power to identify patients at risk.

## 5. Conclusion

EMS staff can use PMEWS ≥ 4 to identify internal patient at risk, especially when they are in doubt about patients' transfer to the hospital. The patients with a very low PMEWS score may still have a life-threatening condition that requires special care in the prehospital environment. Thus, this system should be developed for the support of the EMS staff for decision-making about emergency internal patients in EMS responses. The optimal instrument for detection of emergency internal patient needs more studies to be illuminated.

## Figures and Tables

**Table 1:** PMEWS admission algorithm.

Date:							
Time:							
Patient ID label:							
			Physiological data (MEWS)				

Score	3	2	1	0	1	2	3
Resp. rates	≤8			9–18	19–25	26–29	≥30
O2 Stats.	<89	90–93	94–96	>96			
Heart rate	≤40	41–50		51–100	101–110	111–129	≥130
Systolic BP	≤70	71–90	91–100	>100			
Temp.		≤35	35.1–36	36.1–37.9	38–38.9	≥39	
Neuro.				Alert	Confused/agitated	Voice	Pain unconscious

**Patient data: **Score 1 for each factor							
Age > 65							
Social isolation (lives alone, no fixed abode)							
Chronic disease (respiratory, cardiac, renal, immunosuppressed, and DM)							
**Performance status**				**Score**			
Normal activity without restriction				0	**MEWS:**		
Strenuous activity limited, can do light				1	**Patient data score:**		
Limited activity but capable of self-care				2	**Total PMEWS:**		
Limited activity, limited self-care				3	Name of assessor:		
Confined to bed/chair, no self-care				4	Grade:		

**Table 2:** Patient's characteristics.

Character	N	%
Age > 65	694	32.2
Male	1192	55.3
Female	964	44.7
Social isolation	308	14.3
Chronic disease	1132	52.5
Need of the EMS response	682	68.4
Did not require the EMS response	1474	31.6

	Mean	SD

Age	50.58	22.15
Physiological score	1.97	2.86
Social score	0.75	1.16
PMEWS	2.71	3.55
